# An Integrated Millimeter-Wave Satellite Radiometer Working at Room-Temperature with High Photon Conversion Efficiency

**DOI:** 10.3390/s22062400

**Published:** 2022-03-21

**Authors:** Kerlos Atia Abdalmalak, Gabriel Santamaria Botello, Mallika Irene Suresh, Enderson Falcón-Gómez, Alejandro Rivera Lavado, Luis Enrique García-Muñoz

**Affiliations:** 1 Signal Theory and Communications Department, Carlos III University of Madrid, 28903 Madrid, Spain; efalcon@pa.uc3m.es (E.F.-G.); legarcia@ing.uc3m.es (L.E.G.-M.); 2 Electrical Engineering Department, Aswan University, Aswan 81542, Egypt; 3 Electrical, Computer, and Energy Engineering Department, University of Colorado, Boulder, CO 80309, USA; gasantam@pa.uc3m.es; 4 Department of Physics, University of Otago, Dunedin 3016, New Zealand; mallika.suresh@otago.ac.nz; 5 Yebes Observatory, National Geographic Institute of Spain, 19141 Guadalajara, Spain; arivera@ing.uc3m.es

**Keywords:** radiometers, whispering gallery mode (WGM) resonators, room-temperature receivers, optoelectronic upconversion, high photon conversion efficiency, millimeter-wave radiation, satellite earth observation

## Abstract

In this work, the design of an integrated 183GHz radiometer frontend for earth observation applications on satellites is presented. By means of the efficient electro-optic modulation of a laser pump with the observed millimeter-wave signal followed by the detection of the generated optical sideband, a room-temperature low-noise receiver frontend alternative to conventional Low Noise Amplifiers (LNAs) or Schottky mixers is proposed. Efficient millimeter-wave to 1550 nm upconversion is realized via a nonlinear optical process in a triply resonant high-Q Lithium Niobate (LN) Whispering Gallery Mode (WGM) resonator. By engineering a micromachined millimeter-wave cavity that maximizes the overlap with the optical modes while guaranteeing phase matching, the system has a predicted normalized photon-conversion efficiency $\approx 10^{-1}$ per mW pump power, surpassing the state-of-the-art by around three orders of magnitude at millimeter-wave frequencies. A piezo-driven millimeter-wave tuning mechanism is designed to compensate for the fabrication and assembly tolerances and reduces the complexity of the manufacturing process.

## 1. Introduction

Millimeter-wave and terahertz (THz) radiation detection is critical for many applications such as satellite-based earth observation, radio astronomy, planetary missions, and spectroscopy. For instance, there is a focus on millimeter-wave radiation at 183GHz and 60GHz for water vapor profile measurements and weather forecasting, respectively [1]. Conventional receiver frontends such as, e.g., LNAs and mixers, suffer from an approximately exponential noise performance degradation as frequency increases [2]. Hence, when high sensitivity is needed at millimeter-wave and terahertz frequencies, the receiver frontends are typically operated under cryogenic conditions [3,4], which makes them bulky and expensive [5].

**Figure 1:**
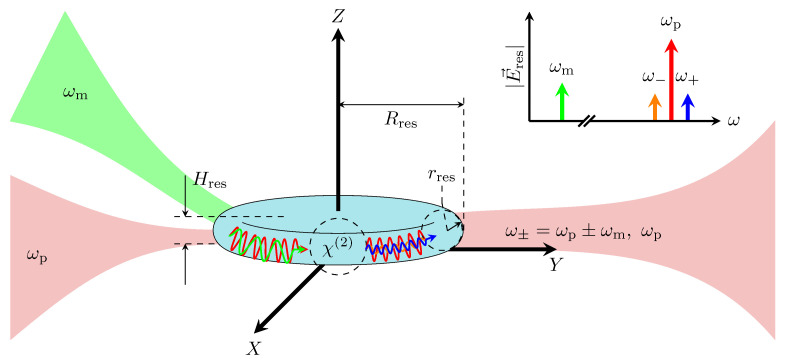
Photonic nonlinear upconversion process.

A different approach for millimeter-wave (or terahertz) detection is to upconvert the radiation to the optical domain, e.g., the telecom band (1550 nm or 193THz), via electro-optic modulation in lithium niobate (LN), which is a medium exhibiting strong Pockels effect. The thermal occupation of optical modes is negligible at room temperature, and low noise detection can be readily achieved via photodetectors with little or no cooling [6]. This significantly reduces the cost, weight, and volume of the receiver and is especially advantageous in satellite radiometers, where the lack of a cryostat increases the satellite mission’s lifetime [7]. Although LN has one of the strongest Pockels effects available in optically transparent media, the second-order susceptibility tensor ($\chi^{\left(2\right)}$) is on the order of tens of pm/V limiting the upconversion efficiency in standard setups. For this reason, high-Q Whispering Gallery Mode (WGM) resonators made of LN have been used to enable higher photon conversion efficiencies [8,9]. If the resonator is designed such that both the millimeter-wave signal and optical pump lie within appropriate resonant modes, their intra-cavity field intensities are enhanced owing to the high-quality factor and finesse of WGMs. This enhances the nonlinear interaction that gives rise to Sum-Frequency Generation (SFG) and Difference-Frequency Generation (DFG) sidebands on either side of the optical pump, as depicted in Figure 1. Here, both SFG and DFG sidebands contain all the information from the input signal. To describe the nonlinear mixing process mathematically in terms of the medium properties and the electric fields involved, the electric polarization of the material, $P(\omega_{+},r)$, at a given position r and frequency $\omega_{+}$ is used. Each vector component is provided as follows:

(1) $$\begin{array}{c} \begin{array}{c} P_{i}\left(\omega_{+},r\right)=\epsilon_{0}\chi_{ij}^{\left(1\right)}\left(\omega_{+}\right)E_{j}\left(\omega_{+},r\right)+\epsilon_{0}\chi_{ijk}^{\left(2\right)}\left(\omega_{+};\omega_{m},\omega_{p}\right)E_{j}\left(\omega_{m},r\right)E_{k}\left(\omega_{p},r\right), \end{array} \end{array}$$

where $\chi^{\left(1\right)}\left(\omega_{+}\right)$ and $\chi^{\left(2\right)}\left(\omega_{+};\omega_{m},\omega_{p}\right)$ are the first and second-order susceptibility tensors of the resonator material, respectively, $E_{j}\left(\omega_{m},r\right)$ and $E_{k}\left(\omega_{p},r\right)$ are the *j*-th and *k*-th vector components of the electric field complex amplitudes associated with the modes at frequencies $\omega_{m}$ and $\omega_{p}$, respectively, and $\epsilon_{0}$ is the permittivity of free space. While the expansion above is valid for a SFG sideband, the DFG sideband has the same form but with one of the electric fields conjugated in the product. Efficient upconversion will only occur under the condition of phase matching, which requires the phase velocity (and angular velocity) of all modes (millimeter-wave, pump, and sidebands) to be equal.

Despite the fact that high-Q resonators significantly improve the photon’s conversion efficiency, the experimentally demonstrated values at millimeter-wave and terahertz frequencies are still low, on the order of $10^{-7}\;{\mathrm{mW}}^{-1}$ pump power [10,11]. Previous experiments showed a record measured normalized efficiency of $2.48\times 10^{-5}\;{\mathrm{mW}}^{-1}$ around 80GHz [12]. Nevertheless, further enhancements in photon conversion efficiency are still needed to make photonic upconversion a good frontend candidate for satellite, radio astronomy, and many other applications [13,14]. Upconversion in WGM resonators is additionally useful for narrowband and single-sideband detection as this is provided naturally by the WGM resonator due to its high-Q and dispersive properties [8].

In [14,15,16], it was shown how room-temperature photonic upconversion receivers could have similar or better noise levels than state-of-the-art cooled millimeter-wave and terhertz LNAs and Schottky mixers [17,18,19,20,21,22] under the condition that absolute photon conversion efficiencies above $10^{-2}$ are accomplished. Even though the benefits are more evident at higher frequencies (above 0.5THz), in this paper, we present a proof-of-concept upconverter around 0.2THz that can be scaled up in frequency with similar performance in a straightforward manner. In particular, we show that the system is robust against the fabrication tolerances of the millimeter-wave and optical components, including the diamond-cut and polished WGM resonator.

## 2. Enhancement of the Upconversion Photon Conversion Efficiency

A figure-of-merit to quantify the quality of the upconverter is the photon conversion efficiency, which is the ratio of upconverted photons per incoming millimeter-wave:

(2) $$\begin{array}{c} \begin{array}{c} \eta =\frac{P_{\pm }/\left(\hbar \omega_{\pm }\right)}{P_{m}/\left(\hbar \omega_{m}\right)}, \end{array} \end{array}$$

where $P_{\pm }$ and $P_{m}$ are the power of the generated sideband (with angular frequency $\omega_{\pm }$) and input millimeter-wave power (with angular frequency $\omega_{m}$), respectively. Using the cavity electro-optics model [23,24] by computing the Hamiltonian of the system with the quantized fields, the photon conversion efficiency can be written as follows [25]:

(3) $$\begin{array}{c} \begin{array}{c} \eta =\frac{g^{2}Q_{p}^{2}Q_{m}}{\hbar \omega_{\pm }\omega_{p}^{2}\omega_{m}}P_{p}, \end{array} \end{array}$$

where $P_{p}$ is the laser pump power with angular frequency $\omega_{p}$, *ℏ* is the reduced Planck’s constant, $Q_{p}$ and $Q_{m}$ are the resonator intrinsic quality factors at optical and millimeter-wave frequency, respectively, and *g* is the nonlinear coupling rate, which has the following form:

(4) $$\begin{array}{c} \begin{array}{c} g=2\chi^{\left(2\right)}\frac{\omega_{p}}{n_{p}^{2}}\sqrt{\frac{\hbar \omega_{m}}{2\epsilon_{0}n_{m}^{2}V_{m}}}E_{m}^{\prime }\left(r_{p}\right), \end{array} \end{array}$$

where $\chi^{\left(2\right)}$ is the second-order susceptibility of the resonator material, $n_{p}$ and $n_{m}$ are the refractive indices for resonator material at optical pump and millimeter-wave frequencies, respectively, $E_{m}^{\prime }\left(r_{p}\right)$ is the normalized millimeter-wave electric field ($E_{m}^{\prime }=E_{m}/E{}_{m}\left(max\right)$) at the location where the optical modes are excited ($r_{p}$), and $V_{m}$ is the mode volume of the millimeter-wave signal. By substituting the nonlinear coupling rate (4) into (3), the photon conversion efficiency can be estimated by using the following equation.

(5) $$\begin{array}{c} \begin{array}{c} \eta =\frac{2P_{p}}{\epsilon_{0}\omega_{\pm }}\frac{\chi^{{\left(2\right)}^{2}}Q_{p}^{2}Q_{m}}{n_{p}^{4}n_{m}^{2}}\frac{E_{m}^{\prime 2}\left(r_{p}\right)}{V_{m}}, \end{array} \end{array}$$

To make fair comparisons of the efficiency between different systems, this value is usually normalized by the continuous wave (CW) pump power ($P_{p}$). As it can be observed from (5), the photon conversion efficiency can be factorized into three terms. The first one cannot be enhanced because it is related to the frequency (application) and pump power. Increasing the pump power using optical amplifiers could lead to problems such as excessive noise in the system and photorefractive effects. Moreover, it is necessary to have strong pump suppression to cleanly detect the upconverted signal.

The second term is related to the material chosen for fabricating the resonator. Several nonlinear materials have been investigated as disk-shaped or ring-shaped WGM resonators such as Gallium Arsenide (GaAs) [26,27], Gallium Phosphide (GaP) [28], Lithium Niobate (LN) [29], Lithium Tetraborate (LT) [30], Quartz [31], and Zinc Selenide (ZnSe) [32]. Extensive comparisons between the properties of the most common nonlinear materials used for the WGM resonators can be found in [11,33]. After analyzing several nonlinear materials and returning to the second term in (5), LN is seen to be a good choice as it has been observed to provide a high optical Q-factor of about $10^{8}$ and an acceptable value for the susceptibility ($\chi^{\left(2\right)}$) of about 150pm/V with reasonable refractive indexes to facilitate achieving the phase-matching condition.

**Figure 2:**
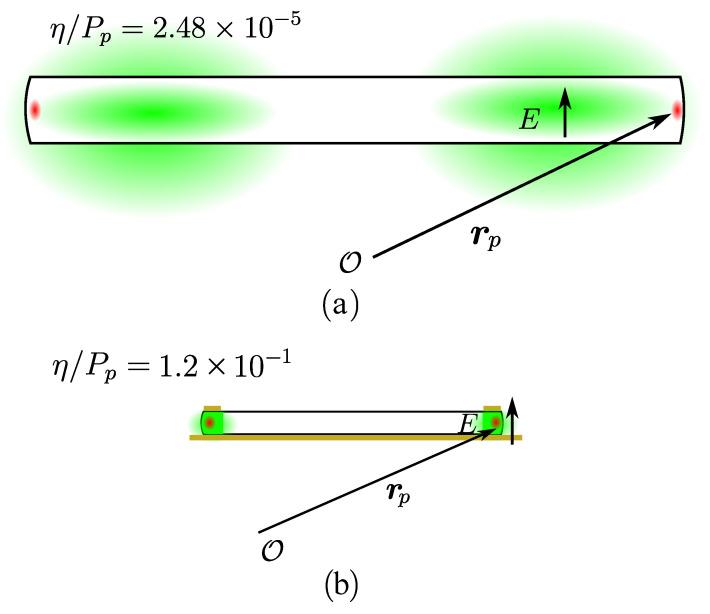
Comparison between millimeter-wave and optical modes for the following: (**a**) a disk resonator; (**b**) the proposed metal-enclosed resonator. Figures not drawn to scale.

Finally, the third term represents the overlap between the two interacting signals, and it mainly depends on the millimeter-wave frequency, the shape of the resonator, and the coupling scheme. Enhancing it means decreasing the millimeter-wave mode volume (in other words, increasing the intensity of the field for given stored energy) and pushing it outwards to the rim of the resonator, where the optical signal is confined. Decreasing the millimeter-wave mode volume will allow more millimeter-wave photons to overlap with the optical ones, enhancing the nonlinear interaction. Due to the large wavelength difference between the two signals—millimeter-wave (some tens or hundreds of GHz) and optical (193.41THz corresponding to a 1550nm laser)—the mode volumes of the signals are orders of magnitude from each other; therefore, there is inherently a very low interaction between the photons. For comparison purposes, Figure 2a depicts the mode volume of the microwave WGM (in green) and the optical WGM (in red) for an experiment using a LN disk resonator as shown in [12]. Although this experiment showed a record measured normalized efficiency at the millimeter-wave range of $2.48\times 10^{-5}\;{\mathrm{mW}}^{-1}$, there is still room for improving the modal overlap.

To modify the millimeter-wave mode volume, the resonator is placed between two metal pieces to confine the millimeter-wave signal to a quasi-TEM mode in a much thinner resonator ($H_{res}$=60μm instead of 350μm in Figure 2a) to be closer to the optical mode size [34], as shown in Figure 2b. It is worth noting here that although using smaller thicknesses would increase the photon’s conversion efficiency (as it is inversely proportional to the millimeter-wave mode volume), it is not recommended to go lower than 60μm. This is because, following our analytical estimations of the spatial distribution of the optical mode [34], it occupies a thickness slightly below 60μm. Thus, decreasing the thickness of the resonator even further would cause the metallic plates to disturb the optical mode and degrade significantly their quality factor. Furthermore, the manufacturing complexity increases drastically, as it becomes more difficult to polish thinner resonators to the required high-Q levels (around $10^{8}$). The upper metal piece can additionally be shaped similarly to a ring to force the millimeter-wave mode outwards to the rim of the resonator and to maximize the $E_{m}^{\prime }\left(r_{p}\right)$ factor and further increase the interaction between the mixing photons.

## 3. Proposed Upconversion Scheme

**Figure 3:**
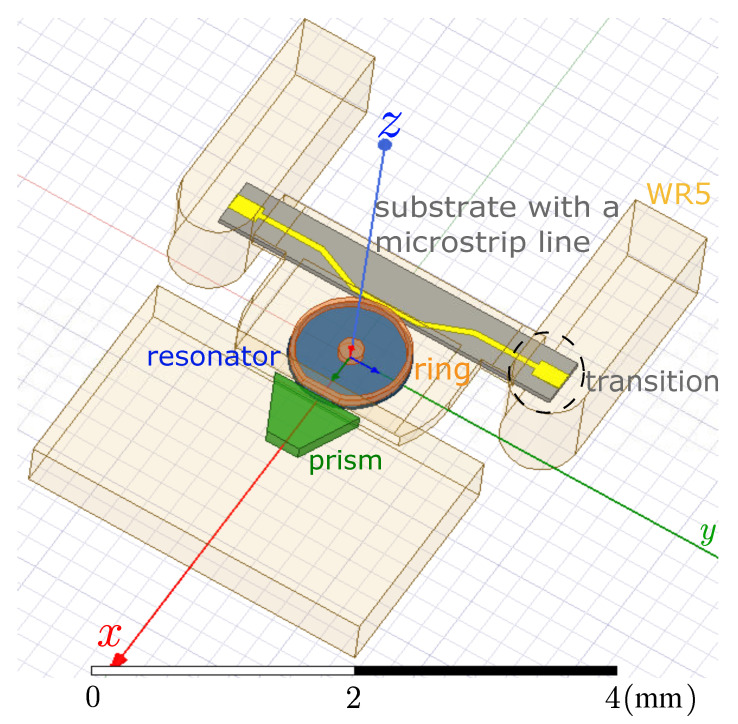
The upconversion scheme with microstrip and prism for millimeter-wave and optical coupling, respectively.

The resonator is a disk made of LN, and its radius ($R_{res}$) optimized to be 488μm to ensure fulfilling the phase-matching condition at 183GHz when a millimeter-wave WGM with angular order m=4 is excited. That is, the mode fits four wavelengths circulating inside the resonator at the intended frequency. Figure 3 presents the proposed WGM upconversion scheme where, as discussed before, the resonator (depicted in blue) is placed between a ground plane and a metal ring (depicted in orange) to increase the photon conversion efficiency of the system. Typically, dielectric rod waveguides are used to efficiently couple radiation into WGM resonators in the millimeter-wave ranges [35,36]. In this design, however, a microstrip line (yellow) is found to be more suitable for the millimeter-wave coupling to keep the confinement of the E-field needed for this metal-enclosed resonator. This microstrip line is connected with a transition to standard rectangular waveguides working at this frequency range (WR5). The light-orange parts represent air boxes; and for the WR5, only the hollow rectangle cross-sections are drawn, while the background is assigned as PEC (Perfect Electric Conductor) by default as a first approximation.

Basically, there are two main techniques for optical coupling: optical waveguides and prisms. Both are based on matching the evanescent field of the WGM and that of the coupler mode [37]. Although optical waveguides can be integrated directly into silicon chips [38], it requires some coupling mechanism between the Si waveguide and the resonator. At this stage, we do not have the possibility of using V-grooves or optical bonding, so only lens terminated fiber would be an option. Aligning the Si waveguide substrate around the resonator and the fiber coupling to the Si waveguide would drastically increase the complexity of our setup. It is, of course, possible to have all optical parts and resonator integrated into a whole so that the alignment between the optical block and the resonator is simplified, but we cannot afford (in terms of time) to develop and debug the assembly at this stage. For the actual experiment configuration of such thin resonators, the optical setup is quite challenging and many gears must be tuned before achieving good optical coupling, Therefore, at this stage of the technology, it would be better to keep working with standard free-space prism coupling for debugging, especially where high levels of coupling can be achieved easily by using GRIN lenses.

Recently, there are other coupling methods based on Subwavelength Grating (SWG) metamaterial that enables coupling to a wide range of diameters and materials [39]. However, special care must be taken with these alternatives. As mentioned above, they may restrict the shape of the resonator or the metal pattern needed to be printed. Both are key characteristics to maximize the overlapping between optical and millimeter-wave modes; therefore, the photon’s conversion efficiency would be strongly affected. This is in addition to the fact that the commonly available optical Q-factor of the integrated LN resonator is still lower than standalone ones, which again would decrease the efficiency.

**Figure 4:**
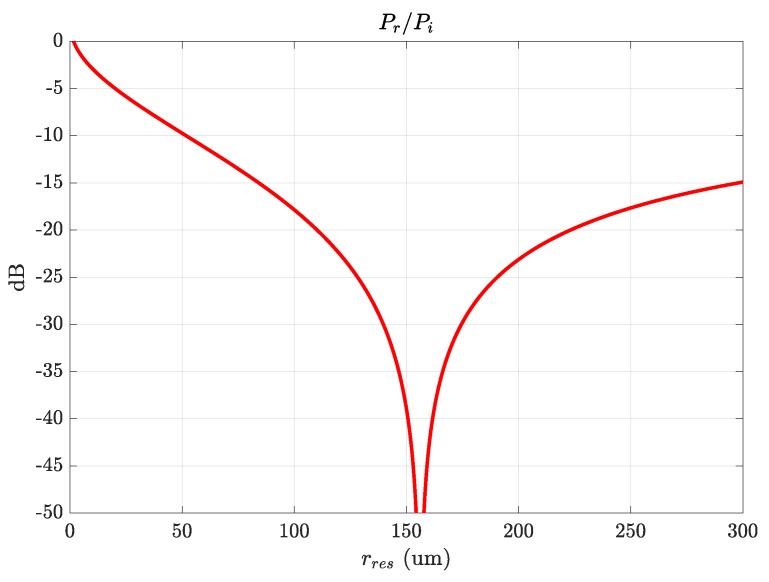
Ratio between reflected optical power to the incident one vs. resonator curvature radius.

Based on this, a diamond prism is used to couple the optical pump coming from the laser, as depicted in green in Figure 3. Two points are considered for increasing the ease of coupling of the optical signal and maximizing intra-cavity power. First, the incidence of the laser light on the prism face is fixed to ($64^{\circ }$) with respect to the normal to the long prism side (X-axis in Figure 3), which is also equal to the prism long-side angles to have a normal incidence of the prism face. This value is determined by simple geometry and depends on the refractive index of the prism $n_{\mathrm{prism}}$ and the effective index of the optical WGM to be excited in the resonator (which is approximated to the material index of the resonator $n_{\mathrm{res}}$), i.e., $\arcsin(n_{\mathrm{res}}/n_{\mathrm{prism}})$. This value is also higher than the critical angle for frustrated total internal reflection from the prism-to-air interface near the resonator. Thus, the evanescent field required for exciting the WGM at the laser frequency is created in the air gap between the prism and the resonator. The height of the prism is set to be 200μm, which is large enough for containing all power from the divergent incident and reflected Gaussian beams but at the same time small enough not to disturb millimeter-wave coupling. Secondly, the resonator has a face curvature ($r_{res}$) of 150μm, which is optimized following our previous calculations in [40], as is shown in Figure 4, to have a negligible reflected optical power.

**Figure 5:**
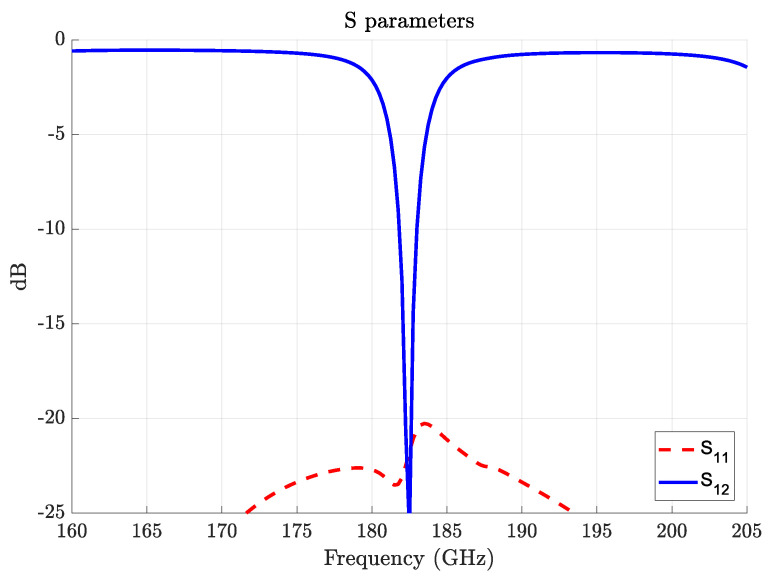
Frequency-dependent S-parameters showing millimeter-wave coupling to the resonator.

Figure 5 depicts the millimeter-wave transmission ($S_{12}$) and reflection ($S_{11}$) coefficients of the upconversion scheme. It demonstrates that the microstrip efficiently couples the millimeter-wave signal at the designed frequency, which ensures that the phase velocity of the millimeter-wave is matched with the optical one. The low value for $S_{12}=-25\;\mathrm{dB}$ at 183 GHz means that most of the power is coupled to the resonator (critical coupling) with less than 1dB lost in the microstrip and waveguide-to-microstrip transitions, as seen from the off-resonance transmission behavior. Likewise, the low $S_{11}$ value means that there is almost no reflection back to the input port, implying that the excited WGM is pure-traveling and not standing-wave. This is desired as a standing-wave WGM could only achieve half of the photon conversion efficiency of a pure traveling one.

**Figure 6:**
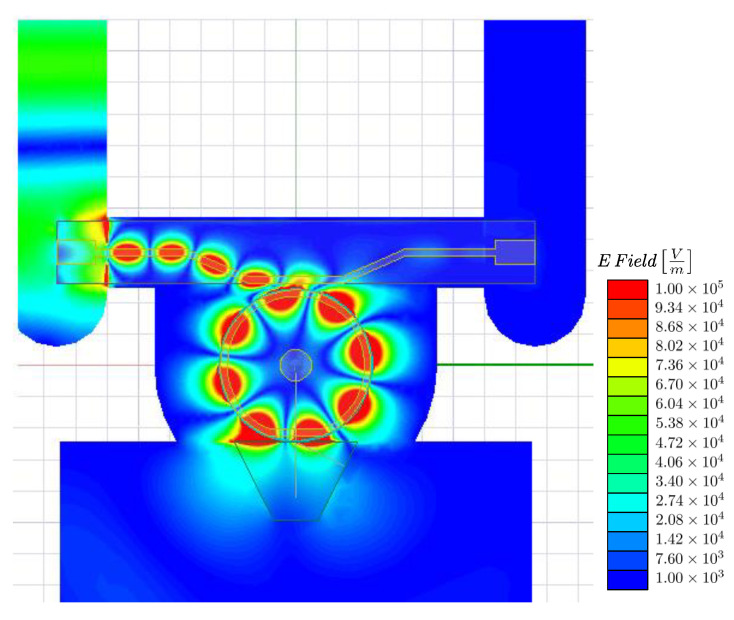
The electric field distribution along the main scheme components with an input power of 1 W.

The electric field distribution is presented in Figure 6 to show the excitation of the required mode (m=4). It shows that the prism has a negligible effect on millimeter-wave coupling as only a small portion of the field overlaps with the structure. Moreover, it shows that the field is well confined at the resonator rim, which increases the overlap between the two signals and, hence, produces higher photon conversion efficiency, as was discussed in Section 2. The predicted photon conversion efficiency is $\eta \approx 1.2\times 10^{-1}\;{\mathrm{mW}}^{-1}$. This provides an enhancement of three orders of magnitude in comparison to our most recent experiment using an LN disk resonator and a dielectric rod waveguide for coupling the millimeter-wave signal into the resonator [12].

**Figure 7:**
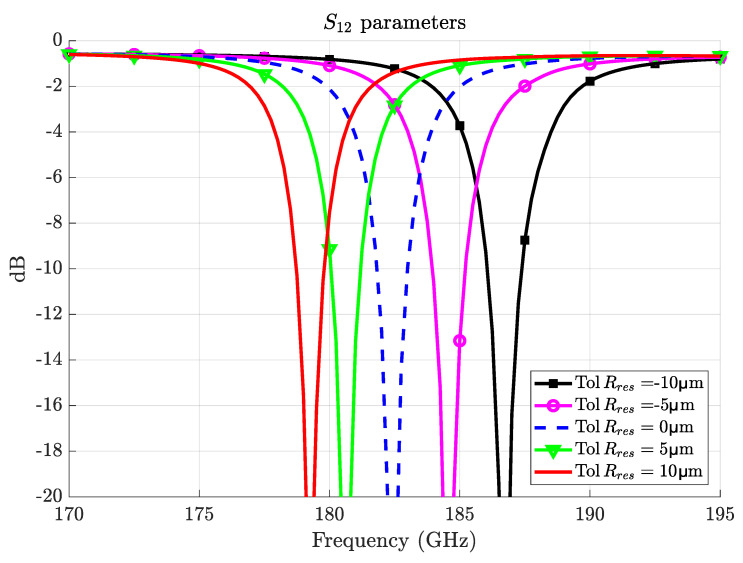
Effect of fabrication tolerances in the resonator radius on resonance frequency.

At millimeter-wave frequencies, fabrication tolerances are one of the main challenges. For example, the LN resonator has a stringent fabrication tolerance of ±10μm and ±2μm for radius and thickness, respectively. The effects of inaccuracy in these dimensions in the millimeter-wave coupling have been simulated as shown in Figure 7 for a variation in radius, which is the dominant source of fabrication tolerance errors.

It can be seen from Figure 7 that a ±10μm deviation in the radius would result in a frequency shift of ±4GHz (while ±2μm thickness errors produce a shift of only ±2GHz). Although this frequency shift is relatively small compared to the resonance frequency (4GHz is around 2%), it is critical for ensuring that the phase-matching condition between the millimeter-wave and optical signals is satisfied.

**Figure 8:**
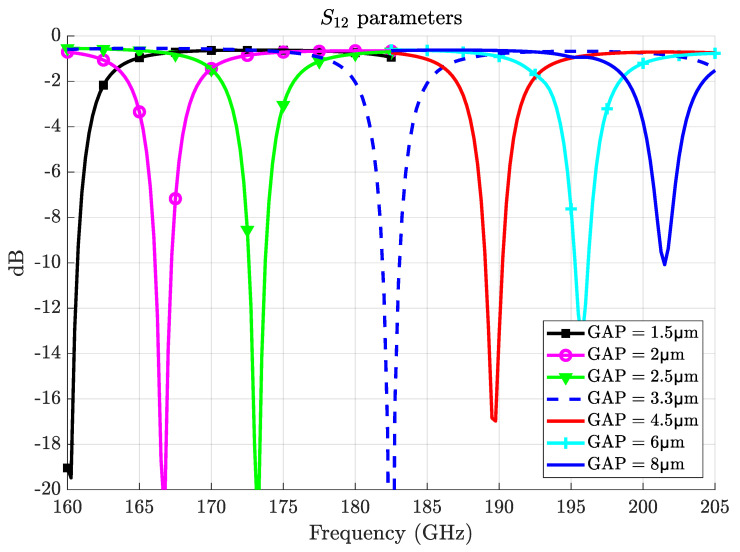
Tuning of the millimeter-wave resonance frequency by using a metallic movable ring.

The design has the advantageous provision for tuning the operating frequency over a wide range to avoid the multiple iterations typically required for achieving phase matching in standard upconversion schemes [25]. As presented in Figure 8, the scheme can efficiently tune the frequency from 160GHz to 205GHz for the same m=4 WGM mode. The tuning is performed by moving the metal ring (as will be shown in Section 4) so that the gap between resonator and ring is varied. In this manner, the effective refractive index exhibited by the millimeter-wave mode is tuned; thus, resonance frequency is controlled. Following this strategy, the upconverter becomes robust against manufacturing tolerances while also circumventing the need for perfect control over material properties as well as environmental conditions that would slightly shift WGM resonances [41]. Additionally, such variations in the gap have a small effect on the system performance as the loaded millimeter-wave Q-factor does not change too much along the entire tuning range (it is in the margin of 85 ± 8).

## 4. Integration of the Upconversion Scheme

**Figure 9:**
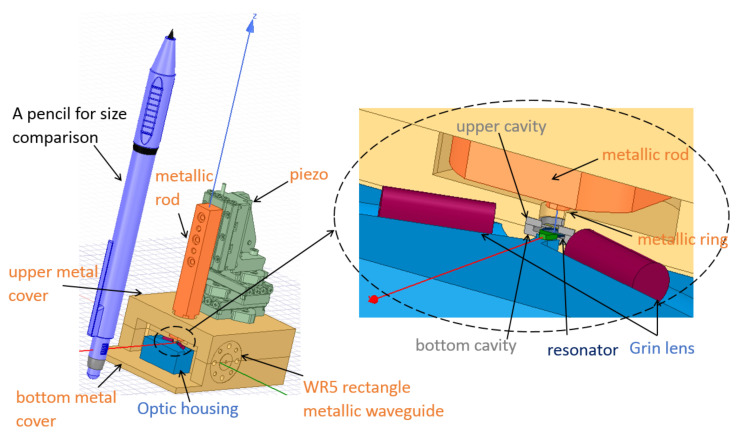
Overview of the integrated system.

Another attractive feature for the proposed scheme is that it can be easily consolidated as a single block as presented in Figure 9. This, along with the small size, weight, and power requirements, makes it suitable for CubeSats and spacecraft-based remote sensing applications. The structure consists of seven main blocks as follows (from bottom to top): bottom metal cover, optic housing, bottom cavity, substrate with a microstrip, upper cavity, rod extension, and upper metal cover. The brown parts are the top and bottom CNC-machined components made of aluminum, which act as an enclosure of the system. They also house the WR5 metallic waveguide in order to be easily connected to the antenna, as shown in the side view of Figure 9.

Inside the metal enclosure and behind the optic housing in blue, the schematic is similar to that shown in Figure 3. The metal ring above the resonator is held with a metal rod (all in orange), which is fixed to a piezoelectric stage from SmartAct, sitting above the upper metal block. To achieve the required fine-tuning discussed in Section 3, the piezoelectric stage is used to move the ring up or down with a precision of 4nm, which corresponds to a frequency shift on the order of tens of megahertz in the resonator. The optical signal comes from a laser and is focused using a GRIN lens (in magenta) towasrd the diamond prism (in green); then, the generated upconverted optical signal is out-coupled using another identical GRIN lens, each symmetrically at $64^{\circ }$ with respect to the long edge of the prism as discussed before. The prism and the two GRIN lenses are glued to the optic housing, which is controlled by another piezoelectric stage (omitted in the pictures for simplicity) to ensure the appropriate prism-resonator gap for optimal optical coupling.

**Figure 10:**
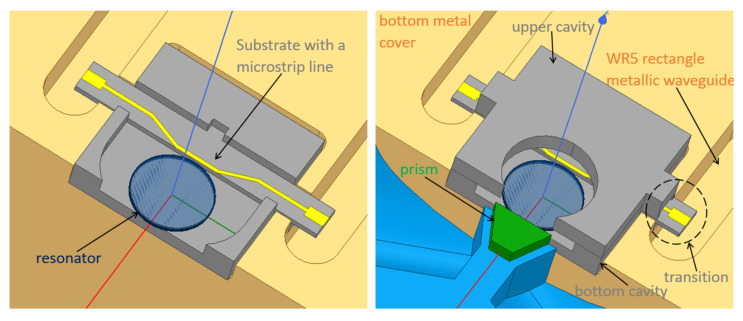
Coupling of the signals into the resonator in the upconversion scheme: left shows the millimeter-wave coupling structure only; right shows both millimeter-wave and optical.

At the back, the parts colored in gray are made out of silicon and are micro-machined then gold-plated (except the substrate on which the microstrip line lies in yellow). They are shown more clearly in Figure 10. They act as the cavity for the resonator and house the microstrip and transition as shown. The hole in the upper cavity is to allow the movable metal ring above the resonator to pass towards or outwards the resonator. Its dimensions are made larger than the ring to allow a slight horizontal tuning of the ring using the 3-axis piezoelectric stage to ensure a good alignment between the ring and the dielectric resonator.

To up-convert higher frequencies such as in the terahertz domain, the proposed scheme can be scaled down. However, in order to not increase the complexity of resonator manufacturing, the same resonator can be still used but by selecting higher-order WGM modes (*m*) to achieve the phase matching at this new frequency. For example, the same resonator can work at 0.5THz or 1THz, but higher-order modes of m=11 and m=22 are needed to be excited at these frequencies, respectively. In this case, the only change would be in the top and bottom covers that contain rectangular metallic waveguides (WR5) that need to be replaced with the corresponding waveguide. In both methods, the performance will remain almost the same since the mode volume and Q of the mode will not change much. This is because the terahertz field is already confined between the metal ring and the ground in a space that much smaller than the wavelength. It is true that the tolerances and misalignment would affect in a higher degree, but tuning can be performed using the metal ring, as discussed in Section 3.

## 5. Conclusions

A compact opto-electronic millimeter-wave receiver for satellite applications has been presented. The scheme is based on the upconversion of the millimeter-wave signal into the optical domain by resonantly enhanced nonlinear optical mixing inside a WGM resonator. The shift to the optical domain eliminates the necessity of cryogenic cooling, which is usually required for the detection of millimeter-wave radiation. The presented system has three main attractive features: First, it has high photon conversion efficiency, outperforming other millimeter-wave upconversion schemes working at room temperature. Second, it is fully integrated and suitable in size, weight, and power requirements for use in satellites. Finally, it has the provision to finely adjust its resonant frequency over a range of ±22GHz, which allows for a compensation of fabrication and alignment tolerances, ensuring the fulfillment of phase-matching conditions. This scheme is not limited to 183 GHz at which this proof-of-concept is presented—in fact, the fine control available to counteract small fabrication inaccuracies encourages extension to higher frequencies.

## Data Availability

Not applicable.
